# Incorporating Ethics in Clinical Guidelines in Infectious Diseases: A Scoping Review

**DOI:** 10.1001/jamanetworkopen.2025.19826

**Published:** 2025-07-10

**Authors:** Dafna Yahav, Asma Nasim, Neta Shirin, Maddalena Armellini, Chiara Zanchi, Varol Tunali, Massimo Mirandola, Aleksandra Barac, Luigia Scudeller, Blin Nagavci, José Ramón Paño-Pardo, Jesús Rodríguez-Baño, Euzebiusz Jamrozik, Murat Akova, Elda Righi

**Affiliations:** 1 Infectious Diseases Unit, Sheba Medical Center, Ramat-Gan, Israel; and Faculty of Medicine & Health Sciences, Tel-Aviv University, Ramat-Aviv, Tel-Aviv, Israel; 2 Department of Infectious Diseases, Sindh Institute of Urology and Transplantation, Karachi, Pakistan; 3 Dipartimento di Diagnostica e Sanità Pubblica, Università di Verona, Verona, Italy; 4 Izmir University of Economics Faculty of Medicine Department of Microbiology, Izmir, Türkiye; 5 Clinic for Infectious and Tropical Diseases, University Clinical Center of Serbia, Faculty of Medicine, University of Belgrade, Belgrade, Serbia; 6 Fondazione IRCCS Policlinico San Matteo, Pavia, Italy; 7 European Society of Clinical Microbiology and Infectious Diseases (ESCMID), Basel, Switzerland; 8 Doctoral School of Clinical Medicine, Semmelweis University, Budapest, Hungary; 9 Servicio de Enfermedades Infecciosas, Hospital Clínico Universitario Lozano Blesa; Departamento de Medicina, Universidad de Zaragoza; Instituto de Investigación Sanitaria Aragón (IISA), Zaragoza, Spain; 10 CIBERINFEC, Instituto de Salud Carlos III, Madrid, Spain; 11 Unidad Clínica de Enfermedades Infecciosas y Microbiología, Hospital Universitario Virgen Macarena; Departamento de Medicina, Universidad de Sevilla; Instituto de Biomedicina de Sevilla/CSIC, Seville, Spain; 12 Monash Bioethics Centre, Monash University, Australia; 13 University of Melbourne, Melbourne, Australia; 14 Department of Infectious Diseases, Hacettepe University School of Medicine, Ankara, Turkey; 15 Guys and St Thomas’s NHS Foundation Trust, London, United Kingdom

## Abstract

This scoping review evaluates whether clinical practice guidelines in infectious disease include ethical considerations.

## Introduction

Infectious diseases raise numerous ethical issues. Yet, incorporating ethics in infectious diseases international clinical practice guidelines (CPGL) remains largely unregulated.^1^ We aimed to evaluate how ethical issues are addressed in CPGL in infectious diseases.

## Methods

This scoping review was conducted in accordance with the Preferred Reporting Items for Systematic Reviews and Meta-Analyses (PRISMA) reporting guideline for scoping reviews. We performed a systematic search for CPGL specific to infectious diseases published between January 2021 and December 2023 in 3 guideline databases. Four researchers (N.S., M.A., C.Z., and V.T.) extracted data from included CPGL characteristics and ethics considerations for guidelines authorship, dedicated ethics sections, approaches to disadvantaged or minoritized populations, costs, justice, access to care, autonomy, public health, and risk-benefit assessment. For study protocol and data extraction manual see eAppendix in Supplement 1.

## Results

Overall, 115 CPGL were included. Characteristics of included CPGL are reported in Table 1. Guidelines authorship (64 [55.7%]), conflict of interest (57 [50.0%]), gender balance (1 [0.9%]), and country balance (2 [1.7%]) policies reporting was limited. Only 32 of 115 CPGL (27.8%) dedicated a section or paragraph to ethical considerations. Minority groups were addressed in approximately half of CPGL (57 [49.6%]), typically by reporting data from available studies (55 [47.8%]). Treatment and diagnostics affordability and costs (53 [46.1%]), access to care (50 [43.5%]), and autonomy (44 [38.3%]) were the most common ethics issues addressed, all in less than half of CPGL. Only 20 CPGL (17.4%) addressed discrimination and 6 (5.2%) discussed liberty.

**Table 1.** Characteristics of the Included Guidelines

Characteristic	Articles retrieved, No. (%) (N = 115)
Year of publication	
2021	44 (38.2)
2022	58 (50.5)
2023	13 (11.3)
Scientific society	
ESCMID	5 (4.4)
IDSA	21 (18.3)
NICE	10 (8.6)
WHO	18 (15.7)
Other^a^	61 (53.0)
Infectious disease topic	
Infective syndromes^b^	19 (16.5)
COVID-19	18 (15.7)
Tropical/neglected disease^c^	18 (15.7)
Hepatitis and STI	17 (14.8)
Bacterial infections^d^	15 (13.0)
HIV/AIDS	9 (7.8)
Hospital-acquired infections	6 (5.2)
Viral infections^e^	5 (4.3)
Other^f^	8 (7.0)
Guideline methodology	
GRADE	49 (42.6)
Not reported	43 (37.4)
Other than GRADE	23 (20.0)
Target population	
General	66 (57.4)
Paediatric	28 (24.3)
Immunocompromised	10 (8.6)
Health care workers	4 (3.6)
Other^g^	7 (6.1)
**Ethical consideration**	
Authorship reporting (n = 115)	
Authorship clearly stated	64 (55.7)
COI reported	57 (50.0)
COI addressed^h^	2 (1.7)
Gender balance policies reported	1 (0.9)
Country balance policies reported	2 (1.7)
Women authors, median, % (IQR)^i^	41.4 (33-50)
Authors affiliated to pharma, median, % (IQR)	7.1 (0-44)
Included authors from LMIC^j^	4 (3.6)
Ethics sections’ content (n = 32)^k^	
Costs and equity	9 (28.1)
Decision sharing, patients’ education, community engagement	9 (28.1)
Patients’ needs-based decision (rather than efficacy)	6 (18.8)
Services tailored to families, specific groups, cultural barriers	5 (15.6)
Specific topic considerations (eg, drug addiction)	3 (9.4)
Reference to minority groups (n = 115)	
Involvement in included trials reported	57 (49.6)
Retrieval of data on minority groups	55 (47.8)
Focused on minority groups^l^	26 (22.6)
Discrimination addressed	20 (17.4)
Ethics issues addressed in guidelines	
Costs	
Diagnostics and treatment affordability	53 (46.1)
Cost or benefit centered on patient	41 (35.7)
Justice or access to care	
Allocation of resources	35 (30.4)
Access to care	50 (43.5)
Patient autonomy addressed	44 (38.3)
Patient liberty assessed	6 (5.2)
Risks and benefits	
Risk/benefit balance assessment	39 (33.9)
Off-label use recognized	8 (7.0)

Abbreviations: COI, conflict of interest; ESCMID, European Society of Clinical Microbiology and Infectious Diseases; GRADE, Grading of Recommendations Assessment, Development, and Evaluation; IDSA, Infectious Diseases Society of America; LMIC, low-middle income country; NICE, National Institute for Health and Care Excellence; RSV, respiratory syncytial virus; STI, sexually transmitted infection; WHO, World Health Organization.

^a^ Other scientific societies are reported in the eAppendix in Supplement 1.

^b^ Chronic otitis media, urinary tract infections, pneumonia, Lyme disease, skin infections, bone and joint infections, cardiothoracic infections.

^c^ Tuberculosis, malaria, schistosomiasis, visceral leishmaniasis.

^d^ *Clostridioides difficile*, multidrug-resistant bacteria, *Helicobacter pylori*.

^e^ Yellow fever, Zika, influenza, RSV.

^f^ Sepsis, vaccination, infections in hemato-oncological patients.

^g^ Women and/or pregnant women, drug users, migrants, patients with medical comorbidities, mixed populations.

^h^ Exclusion of panel member from guidelines or selected recommendations depending on COI.

^i^ Extracted from 56 guidelines.

^j^ Extracted from 59 guidelines.

^k^ Only 32 of 115 guidelines (27.8%) had an ethics section (32 was used as denominator).

^l^ These included young children (35.8%); women (including pregnant women, 32.1%); chronically ill, disabled, or elderly patients (27.8%); and others (4.3%).

Suggestions to address cost issues included the proposal of differential costs according to country and addressing costs in CPGL recommendations (Table 2). Regarding resource allocation and access to care, potential solutions included training, strengthening of laboratories and infrastructure, and offering alternatives where resources are limited. Discussion with patients and family involvement were advocated to promote autonomy, and incorporating patients’ views where appropriate was advised to promote ethically acceptable restrictions of individual liberty (eg, isolation).

**Table 2.** Ethical Issue Details and Proposed Solutions in Guidelines

Ethical issues	Issue details (No. [%]) (N = 115)	Proposed solutions
Costs (affordability; patient cost-benefit analysis)	Lack of drugs in resource limited areas (40 [34.8]) High cost of tests, PPE, hand hygiene monitoring, vaccine, or informatic tools (29 [25.2]) Cost-effectiveness of different regimens (options limited by cost or insurance availability) (17 [14.8]) Subpopulations at high risk not able to afford costs (17 [14.8]) Indirect costs (disease related income loss, hospital stay) (12 [10.4])	Different costs in different countries, negotiations Oral therapy or OPAT Reduce unnecessary treatment Offer alternatives if costs too high Cost-benefit analysis Generic drugs, shorter treatments Point of care testing Scale up centered services Consider costs in each recommendation
Justice or access to care (allocation of resources; access to care)	Means in general (36 [31.3]) Treatment (30 [26.1]) Diagnostics, equipment, material (23 [20.0]) Community services (13 [11.3]) Hospital capacity (7 [6.1]) Workforce (7 [6.1]) Diagnostics (37 [32.2]) Facilities (37 [32.2]) Medication (25 [21.7]) Stigma (12 [10.4]) Information (5 [4.3])	Optimize screening Vaccination in rural areas and for vulnerable populations Allocation of equipment for IPC Identify gaps and inform models of care Prioritize antibiotics Support training Offer alternatives if limited resources Implement infrastructures and laboratories Access to dedicated funding for LMIC Patient centered policies Free of charge care
Autonomy (patient autonomy addressed)	Right to information (52 [45.2]) Refusal and decision making (23 [20.0]) Choice of health care facility (17 [14.8]) Referral of care (12 [10.4]) Privacy and confidentiality (12 [10.4])	Risk and benefit discussion with patients OPAT Education and multilingual leaflets Choice discussion with patients Provide updated information on safety and adverse effects Information about own health Involvement of families Patients’ active contribution in the decision Informed consent obtained and shared decision making MDT discussion Safeguard privacy Identify vulnerable groups Cultural and religious beliefs considered Confidentiality, empathy and a nonjudgemental approach Involve affected communities in the design and implementation of health care centers Sharing goals DOT
Public health (patient liberty assessed)	Isolation or contact precautions (35 [30.4]) Involvement in decision (35 [30.4]) Privacy (23 [20.0]) Education (23 [20.0])	Invite patients to comment on recommendations Right to refuse

Abbreviations: DOT, directly observed therapy; IPC, infection prevention and control; LMIC, low-middle income country; MDT, multidisciplinary team; OPAT, outpatient parenteral antimicrobial therapy; PPE, personal protective equipment.

## Discussion

The explicit incorporation of ethical issues in infectious disease CPGL is limited. The aspects of ethics in treating infectious diseases are intrinsically multifaceted and often involve economically or socially disadvantaged populations, with implications for public health, resource allocation, discrimination, patient autonomy, justice, and more.^2^ The majority of retrieved CPGL came from high-income countries. Furthermore, infectious disease CPGL may present inadequate reporting of authorship and conflicts of interest, with underrepresentation of female and LMIC authors, despite addressing relevant topics (tuberculosis, HIV). Less than a third of CPGL dedicated a section or paragraph to ethical considerations, and only half of guidelines addressed minority populations. The most common ethical issues addressed were related to justice (including affordability and access to care). Complex ethical issues apply to the field of infectious diseases because of disproportionate disease burdens in vulnerable populations and the public health risks related to the spread of infection. Nevertheless, current literature regarding implementation of ethical aspects in infectious disease CPGL is limited. Strategies for inclusion of ethical issues in CPGL in general include defining disease-specific ethical issues (DSEI) and appropriate planning of CPGL development (eg, by including panel members with ethics expertise, incorporating the results of patient or public engagement, addressing the needs of vulnerable populations, and/or defining ethical issues as part of specifying outcomes).^3,4,5,6^ Limitations of this study is lack of quality assessment of included guidelines, as well as the subjectivity in data extraction concerning ethical issues.

Our study reveals that such planning and actual consideration of ethical issues in infectious disease CPGL are limited. This should arguably be improved, for example via guidance for incorporating ethics into future infectious disease CPGL and/or the development of a predefined checklist to ensure appropriate handling of ethical issues.

## Notes

Supplement 1. **eAppendix.** Study Protocol and Supplementary Documentation

Supplement 2.
Data Sharing Statement

## References

[1] Rogers WA. Are guidelines ethical? Some considerations for general practice. Br J Gen Pract. 2002;52(481):663-668. 12171228 PMC1314388

[2] Righi E, Yahav D, Nasim A, ; ESCMID OPENING Project Group. Incorporating ethics into infectious disease clinical practice guidelines. Clin Microbiol Infect. 2025;31(4):560-567. doi:10.1016/j.cmi.2025.01.008 39827993

[3] Sanabria AJ, Kotzeva A, Selva Olid A, . Most guideline organizations lack explicit guidance in how to incorporate cost considerations. J Clin Epidemiol. 2019;116:72-83. doi:10.1016/j.jclinepi.2019.08.004 31430507

[4] Shaver N, Bennett A, Beck A, . Health equity considerations in guideline development: a rapid scoping review. CMAJ Open. 2023;11(2):E357-E371. doi:10.9778/cmajo.20220130 37171906 PMC10139082

[5] Mertz M, Strech D. Systematic and transparent inclusion of ethical issues and recommendations in clinical practice guidelines: a six-step approach. Implement Sci. 2014;9:184. doi:10.1186/s13012-014-0184-y 25472446 PMC4265426

[6] Thomas OP. A discussion of the ethics of clinical guidelines. J Eval Clin Pract. 2019;25(6):980-984. doi:10.1111/jep.13264 31414522

