# Supplementary material for: Incorporating Ethics in Clinical Guidelines in Infectious Diseases: A Scoping Review
Source: JAMA Netw Open. 2025 Jul 10;8(7):e2519826. doi: 10.1001/jamanetworkopen.2025.19826 (PMC12246872; doi:10.1001/jamanetworkopen.2025.19826)
Supplement: Supplement 1. — eAppendix. Study Protocol and Supplementary Documentation [file jamanetwopen-e2519826-s001.pdf]

Supplemental Online Content

Yahav D, Nasim A, Shirin N, et al. Incorporating ethics in clinical guidelines in infectious diseases. *JAMA Netw Open.* 2025;8(7):e2519826. doi:10.1001/jamanetworkopen.2025.19826

eAppendix. Study Protocol and Supplementary Documentation

This supplemental material has been provided by the authors to give readers additional information about their work.

© 2025 Yahav D et al. *JAMA Network Open*.

Protocol

IncOrPorating Ethics iN ClINical Guidelines (OPENING study): a scoping review

This scoping review was conducted as part of a broader project aiming to review current status of ethical considerations in Infectious Diseases/Clinical Microbiology (ID/CM) clinical practice guidelines (CPGL). The project was initiated by the ESCMID ethics advisory committee (EEAC). The aim of the entire project is to use the data collected to build a guidance document defining the standards for systematic inclusion of ethics in the development of ESCMID CPGL.

We provide below to the protocol for the scoping review titled above.

1. Objective

Perform a scoping review evaluating the reporting of ethical principles and issues in CPGL in ID/CM published in recent years.

2. Background

Incorporating ethics in infectious diseases (ID) clinical guidelines remains largely not regulated and current manuals for CPGL development are silent on this point.

An "ethical consideration" session is usually lacking in clinical guidelines. In recent years, however, increasing attention was shown for ethical topics in ID especially with the advent of the COVID-19 pandemics. Various topics in ID deserve ethical considerations while incorporated into guidelines. One example is represented by the availability of resources or drugs (e.g., novel anti-infectives) that may highly vary across countries due to organisational and/or economic disparities. Diagnostics tools' availability in clinical microbiology (CM) may also be affected by costs or lab equipment.

An example for an ethical section in ID/CM is presented in the recent ESCMID guidelines on the treatment of MDR GNB (Paul M et al. CMI 2022). These guidelines included a paragraph on "considering costs and equity" reporting that "…antibiotics are not equally available worldwide. In low-resource settings, costs of the new antibiotics might prohibit their use; and even in other settings costs of the new antibiotics enter the consideration for use. Antibiotics critical to management of the priority pathogens should be defined and more efforts should be implemented to ensure the universal availability of these antibiotics. Differential costs of the new antibiotics for different socio-economic settings will allow better equity of patient management".

Articles addressing how to include ethical consideration in guidelines are scarce and, interestingly, they focus on development of statements on which to base ethical recommendations [Mertz M, Strech D Implem Science 2014] and key steps to include equity-focused reviews [Dewidar O et al J Clin Epidemiol 2022].

© 2025 Yahav D et al. *JAMA Network Open*.

How often and to which extent ID/CM CPGL address consideration of ethical issues has not been evaluated before. In this scoping review we aimed to address this question.

3. Scope

A systematic search of CPGL databases to search how ethical principles are reported in ID/CM guidelines.

4. Methods

Search in CPGL documents for ethical considerations

Systematic searches of ID/CM-specific CPGL, to assess ethical considerations, will be undertaken in three guideline databases: GIN international guideline library, Guideline Central, and Trip medical database (1-3), from January 2021 to December 2023. This time frame was chosen to include guidelines covering a period of three years to be representative of different Infectious Diseases topics, including periods characterised by COVID-19 pandemic and those approaching the end of the pandemic phase.

All records found during these time limits will be manually screened by title/abstract directly in the guideline database to identify CPGLs fulfilling the following criteria:

1. CPGLs on any ID topic.
2. CPGLs on any intervention (diagnostic, treatment, or prognostic).
3. CPGLs regarding any population (no age limits).
4. CPGLs published during 2021-2023
5. CPGLs published in English language.

Potentially relevant records will be screened by full text for eligibility by four researchers (NS, MA, CZ, VT). CPGL fulfilling the above criteria will be included. Data from these will be extracted to an Excel table, including guideline characteristics (title, publication year, publisher, country involvement and World Health Organization (WHO) country areas balance, topic/intervention); and ethics considerations (see below). We will focus on how ethical considerations were addressed in the guidelines. Data will be extracted on transparency in authorship of guidelines, equity in authorship, presence of a dedicated section/paragraph on ethics in the guidelines, data on vulnerable populations /minorities, issues regarding costs, justice and access to care, autonomy, public health, research ethics and risk/benefit assessment. These topics were collected after discussions between the EEAC members and an ethics specialist (EJ). The detailed manual used for data extraction is provided in this Supplemental Appendix below.

© 2025 Yahav D et al. *JAMA Network Open*.

The searches will be conducted and reported in accordance with the Preferred Reporting Items for Systematic reviews and Meta-Analyses (PRISMA) extension for scoping reviews (PRISMA-ScR) [Tricco et al. Ann Intern Med. 2018 Oct 2;169(7):467-473] and based on our internal protocol.

Identification of relevant CPGLs:

Screening of records will be performed in two phases (title/abstract and full text). Title/abstract screening of CPGL will be done by one reviewer directly in the guideline databases. Potentially relevant CPGL will be listed and will be assessed in full text by two reviewers independently.

Extraction of study data:

Relevant data will be extracted in Excel for subsequent analysis, such as CPGL title, publication year, publisher (including societies that involve all ID topics in guidelines, such as IDSA, ESCMID, WHO, NICE or societies/groups focused on specific topics only, reported as "other"), country involvement and WHO country areas balance, topic/intervention, type of ethical topic covered according to the type of ID area and how they are incorporated into the CPGL (e.g., structured inclusion; scattered/sporadic). Ethical considerations will include topics such as resources/drugs distribution, discrimination, equity, and access to care.

Risk of bias:

Risk of Bias is not part of a scoping review and will not be assessed.

Data analyses/summary:

Relevant documents and CPGL will be collected and assessed narratively. Data will be presented using tables. The total number of CPGL retrieved and the number of those addressing ethical issues by topic will be presented.

References:

1. Guidelines International Network (GIN) at https://guidelines.ebmportal.com/ (last accessed 16 Apr 2025)

2. Guidelines Central at https://www.guidelinecentral.com/guidelines/ (last accessed 16 Apr 2025)

3. Trip medical database at https://www.tripdatabase.com/(last accessed 16 Apr 2025)

© 2025 Yahav D et al. *JAMA Network Open*.

Manual for extraction table – ethics in guidelines

Variable	Description
**Identifiers**	
Author, Year, DOI, Organization.	If no DOI is available, add URL.
**Characteristics of GL**	
Topic, Type, Theme, Method, Target population	Add characteristics
**Transparency in authorship**	
Is an authorship clearly stated?	Are names of authors reported, or indicate if unclear
Is there a clear statement about COI and Pharma Company?	Check if authors declared their conflict of interest - COI (regardless of if COI present or not). Focus is declaration of COI and not COI itself.
No of authors	Total number
No of women authors:	To assess the gender of authors, binary gender assignment will be conducted. If first-name assessment and descriptors (he/him, she/her) on manuscript are not clear in terms of gender determination, we will use the https://genderize.io/ application - if the probability is over 0.8 the application is good enough. If it is less, we will use hospital web pages or press releases; personal web pages; Google, Google scholar, ResearchGate, LinkedIn search for images or other descriptors.
No with Pharm affiliation	Number of authors who are affiliated to pharmaceutical companies. Check their COI
L/LMIC authors (see LMIC list)	According to the list of the world bank at https://datatopics.worldbank.org/world-development-indicators/the-world-by-income-and-region.html or at https://qesclimatejustice.info.yorku.ca/resources/eligible-countries/ Consider as L/LMIC the countries reported as low and lower middle income
Is a gender policy reported in text or referred to in the appendix?	Is there a text addressing women/men balance regarding to authors of the guidelines yes/no
Is a country balance policy reported in text or referred to in the appendix?	Is there a text addressing L/LMIC/high income countries balance regarding to authors of the guidelines yes/no
**Structure**	
Is there a dedicated ethics consideration section in GL?	Is there a separate section or at least paragraph addressing specifically ethics in the guidelines? Do not consider ethics generic declaration at the end of the manuscript or scattered considerations on ethics.
If YES, how is it report it (briefly describe)	For example, report the summary of a dedicated paragraph
**Vulnerable populations/minorities**	
Mentioned if trials evaluated in GL included minorities?	Do not check primary studies (trials). Check only the guideline manuscript and appendix for this information.
Are these GL addressing minorities?	Only report if minorities are the focus of the guideline. If more than one minority group is the focus of the guidelines, this will be discussed

© 2025 Yahav D et al. *JAMA Network Open*.

Do GL target mainly/only minorities?	See comment above
What is the main minority group mentioned?	A dropdown list is provided for the type of minority. Leave blank if does not apply.
Specify second/third minority group	Leave blank if does not apply
Is discrimination addressed?	Search for discrimination as a keyword. Report only if guideline clearly address discrimination and not inferred
Are means/diagnostics/drugs cost and affordability addressed?	This means the cost of recommended treatment/tests/care has been addressed by the guidelines. This can be in the form of a statement recognizing the recommended treatment/ test/care being expensive. Search for cost/price/expensive as keywords
Is cost vs. benefit addressed?	Report only if the guideline clearly states what is the benefit of a test or drug; for example: a new rapid test for MRSA is expensive but would allow to reduce MRSA infections in ICUs in LMIC, therefore GL suggest that differential cost for different countries should be considered
Are benefits or cost reduction addressing patients (patient centred)?	Report only if the guideline clearly states what that the benefits are for the patients
**Justice/Access to care**	
Is allocation of resources addressed?	Distributive justice refers to the fair, equitable, and appropriate distribution of healthcare resources. Should be clearly stated and not inferred. Most guidelines may not address this issue. Do the guidelines refer to this issue – yes/no
Type of ethical issue (allocation)	Choose from a dropdown list
Greater good issues (utilitarianism)	May be found in guidelines on COVID and healthcare workers or even antimicrobial resistance. Only add it if there are sentences clearly stating that a certain indication or intervention aims at promoting the greatest good for the greater amount of people without focusing on the single person – e.g., antibiotic over prescription for the single patient does not promote greater good; physicians during COVID-19 refusing to care for COVID-19 do not promote greater good.
Is access to care addressed?	Use words from the dropdown list as keywords. This means the availability for everyone (irrespective of age, economic capacity, health insurance for US guidelines, etc.) of the recommended treatment/test/care has been addressed or not.
Type of ethical issue (access)	Use dropdown list; if more than one, choose the most relevant access ethical issue; for example: a guideline may address having point of care tests in all rural ambulatories, otherwise people cannot test unless living in the city.<br>If there is more than one access to care issue the topic ill be discussed within the group
Describe solution for access if suggested (briefly)	
**Autonomy**	

© 2025 Yahav D et al. *JAMA Network Open*.

Is patient autonomy addressed?	This means whether there is any statement in which patients concerns/preferences have been addressed? Does the guideline mention any concerns on behalf of patients for example statement from patients or any alternative suggestions if patients refuse the recommended treatment/test/care? Use words from the dropdown list as keywords.
Type of ethical issue (autonomy)	Choose from a dropdown list
Is a solution proposed (autonomy)?	Yes/No
Describe solution (autonomy)	Report briefly any statement highlighting its solution is present in the guidelines or not
**Public health**	
Is patient liberty addressed?	This may be expected for COVID-19 guidelines addressing liberty e.g., lockdown or articles addressing isolation or quarantine. Use words from the dropdown list as keywords
Type of ethical issue (liberty)	Dropdown list
Other issue specify (liberty)	Yes/No
Is a solution proposed (liberty)?	Yes/No
Describe solution (liberty)	Report briefly any statement highlighting its solution is present in the guidelines or not
**Research Ethics**	
Is research ethics addressed?	Only consider this point if issues mentioned in the dropdown list are reported. For example, if a COVID-19 guideline mentions that there are concerns regarding peer review and they decided to include or exclude evidence from these articles.
Type of issue (research)	Dropdown list
Other issue specify (research)	Yes/No
Is a solution proposed (research)?	Yes/No
Describe solution (research)	Report briefly any statement highlighting its solution is present in the guidelines or not
**Risk/benefits**	
Risk/benefits balance addressed (allergies, toxicity, etc.)	This means any statement discussing risks and benefits for adverse effects of the recommended treatment/test /care and how it will be addressed or quality of life or any statement addressing breach of patient privacy and how it will be addressed. Only report YES if this point is clearly addressed in the guidelines and not just mention of the fact that certain drugs can give toxicity.
Off label use recognized	Report only if off label use is discussed in the guidelines (use off-label as a keyword)

© 2025 Yahav D et al. *JAMA Network Open*.

PRISMA Flow-chart

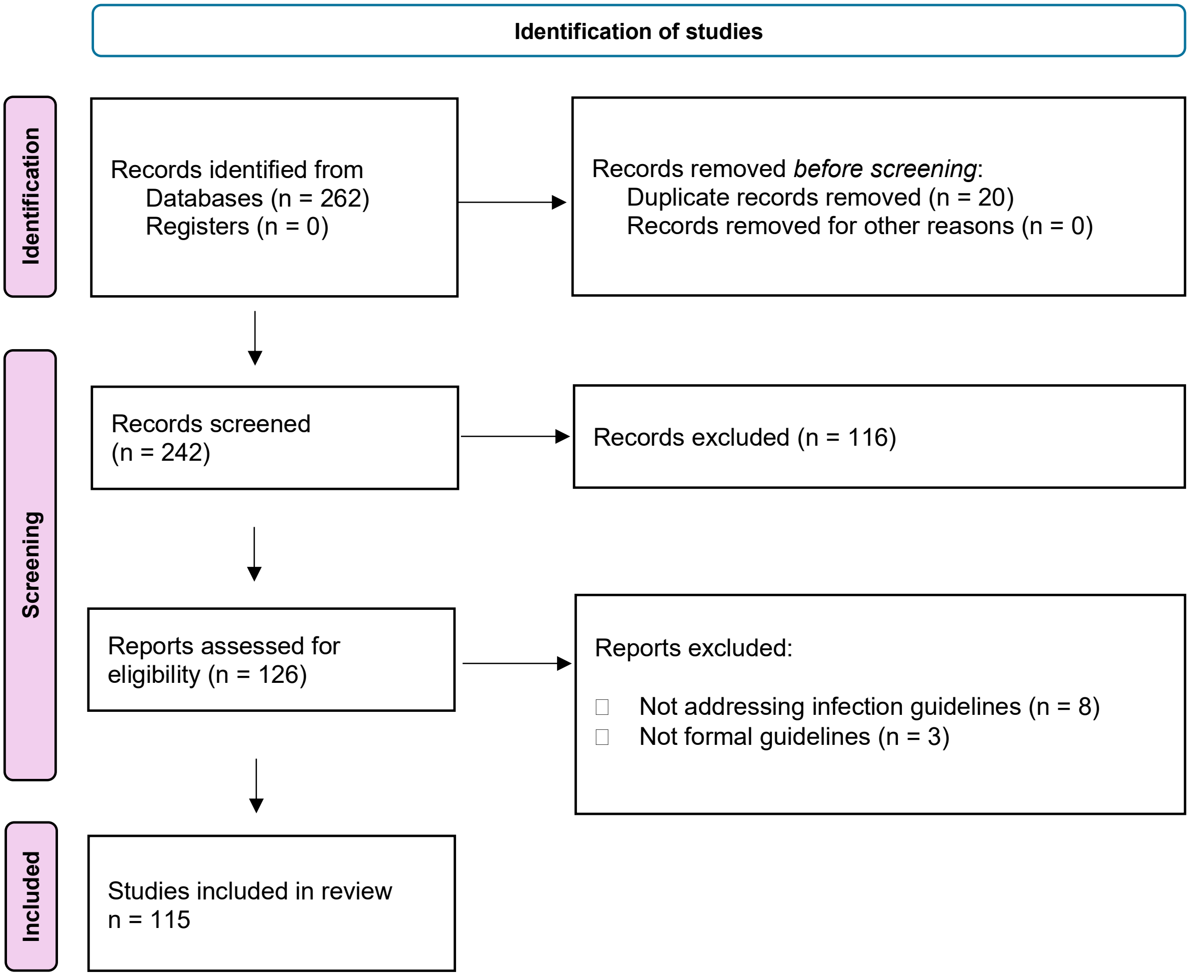

© 2025 Yahav D et al. *JAMA Network Open*.

Scientific societies other than ESCMID, IDSA, NICE, WHO and associated countries of origin.

These were defined as societies that may not consistently publish on all ID topics.

Other societies included:

- British Association for Sexual Health and HIV (BASHH), United Kingdom
- Canadian National Advisory Committee on Immunization (NACI), Canada
- American Heart Association (AHA), United States
- Brazilian Society of Rheumatology (BSR), Brazil
- Canadian Thoracic Society, Canada
- Infectious Diseases Working Party (AGIHO) of the German Society for Hematology and Medical Oncology (DGHO), Germany
- Italian Society of Infection and Tropical Diseases (SIMIT)/Italian Society of Anti-Infective Therapy (SITA), Italy
- Australian diabetes related foot disease guidelines and pathways project, Australia
- European Conference on Infections in Leukaemia (ECIL), Europe
- German Association of the Scientific Medical Societies, Germany
- Japan Otological Society and Oto-Rhino-Laryngeal Society of Japan, Japan
- Centre of Research Excellence in Ear and Hearing Health of Aboriginal and Torres Strait Islander Children, Australia
- World Gastroenterology Organisation, multiple countries
- The National Prisons Hepatitis Network (NPHN), Australia
- American College of Obstetricians and Gynaecologists (ACOG), United States
- SHEA (The Society for Healthcare Epidemiology of America) , United States
- Spanish Society of Cardiovascular Infections (SEICAV), the Spanish Society of Thoracic and Cardiovascular Surgery (SECTCV) and the Biomedical Research Centre Network for Respiratory Diseases (CIBERES), Spain
- Canadian Paediatric Society, Canada
- American Academy of Orthopaedic Surgeons (AAOS) , United States
- US Preventive Services Task Force (USPSTF) , United States
- Gastroenterological Society of Australia (GESA), Australia
- European Society of Intensive Care Medicine (ESICM)/Society of Critical Care Medicine (SCCM), Europe
- European Centre for Disease Prevention and Control, Europe
- Infectious Diseases Working Party of European Society of Blood and Marrow Transplantation/International Immunocompromised Host Society/European Leukemia Net, Europe
- National Clinical Effectiveness Committee (NCEC), Ireland
- Association of Paediatric Anaesthetists of Great Britain and Ireland, United Kingdom and Ireland
- Guidelines and Protocols Advisory Committee on behalf of the Medical Services Commission, Canada
- Australian and New Zealand Children’s Haematology/Oncology Group, Australia and New Zealand
- The Children's HIV Association, United Kingdom

© 2025 Yahav D et al. *JAMA Network Open*.
