# Supplementary material for: Incorporating Ethics in Clinical Guidelines in Infectious Diseases: A Scoping Review
Source: JAMA Netw Open. 2025 Jul 10;8(7):e2519826. doi: 10.1001/jamanetworkopen.2025.19826 (PMC12246872; doi:10.1001/jamanetworkopen.2025.19826)
Supplement: Supplement 2. — Data Sharing Statement [file jamanetwopen-e2519826-s002.pdf]

Data Sharing Statement

Yahav. Incorporating Ethics in Clinical Guidelines in Infectious Diseases. *JAMA Netw Open*.
Published July 10, 2025. doi:10.1001/jamanetworkopen.2025.19826

Data
Data available: Yes
Data types: Other (please specify)
Additional Information: Data available upon request
How to access data: Dr. Elda Righi at eldarighi36@gmail.com
When available: With publication

Supporting Documents
Document types: None

Additional Information
Who can access the data: anyone requesting the data
Types of analyses: All data
Mechanisms of data availability: Correspondense with Dr. Righi
Any additional restrictions: None
